# Drug utilisation in neonatal units in England and Wales: a national cohort study

**DOI:** 10.1007/s00228-021-03267-x

**Published:** 2022-01-13

**Authors:** Asma Al-Turkait, Lisa Szatkowski, Imti Choonara, Shalini Ojha

**Affiliations:** 1 Academic Unit of Population and Lifespan Sciences, School of Medicine, University of Nottingham, Nottingham, NG7 2RD UK; 2 Neonatal Unit, University Hospitals of Derby and Burton NHS Trust, Derby, UK

**Keywords:** Drug utilisation, Rational prescribing, Antibiotics

## Abstract

**Purpose:**

To describe drug utilisation patterns in neonatal units.

**Methods:**

Retrospective observational cohort study using data held in the National Neonatal Research Database (NNRD) for neonatal units in England and Wales including infants born at 23 to 44 weeks’ gestational age (GA) from 01 January 2010 to 31 December 2017.

**Results:**

The cohort included 17,501 (3%) extremely preterm infants; 40,607 (7%) very preterm infants; 193,536 (31%) moderate-to-late preterm infants; and 371,606 (59%) term infants. The number of unique drugs received by an infant (median (IQR)) increased with decreasing GA: 17 (11–24) in extremely preterm, 7 (5–11) in very preterm, 3 (0–4) in moderate-to-late preterm, and 3 (0–3) in term infants. The two most frequently prescribed drugs were benzylpenicillin and gentamicin in all GA groups, and caffeine in extremely preterm. Other frequently used drugs among preterm infants were electrolytes, diuretics and anti-reflux medications. Among infants <32 weeks’ GA, the largest increase in use was for surfactant (given on the neonatal unit), caffeine and probiotics, while domperidone and ranitidine had the largest decline.

**Conclusion:**

Antibiotics, for all GAs and caffeine, among preterm infants, are the most frequently used drugs in neonatal medicine. Preterm infants are exposed to a high burden of drugs, particularly antibiotics. Changing patterns in use reflect the emergence of evidence in some areas but several non-evidence-based drugs continue to be used widely. Improvements are needed to ensure rational drug use on neonatal units.

**Registration:**

ClinicalTrials.gov (NCT03773289). Date of registration 21 Dec 2018.

**Supplementary information:**

The online version contains supplementary material available at 10.1007/s00228-021-03267-x.

## Introduction

Drug utilisation studies highlight aspects such as pattern, variability and trends in pharmacotherapy. They inform design and implementation of effective strategies for rational prescribing practices and inform research [1]. While there are several small studies describing patterns of drug utilisation in neonatal units, very few have reported national pictures or evaluated drug use over longer periods [2].

In the UK, a survey of 49 neonatal units evaluated drug use over a 2-week period in 2007–2008 [3]. It provided some insight into the agenda for medicine research in the UK. However, it included an arbitrary two-week period of data collection that varied between participating units and had a low response rate. Large scale drug utilisation studies from the USA [4, 5] have described prescribing patterns and change over time from selected centres. No such studies have been conducted in the UK where the provision of neonatal care is almost exclusively within the National Health Service (NHS), providing a unique opportunity to evaluate drug use at a population level.

Patterns of drug utilisation have been widely reported in other settings [2]. Their results inform clinical practice including therapeutic protocols and guideline development. Information about evolving patterns of change in drug use represent adoption of new practices based on emerging evidence. Such studies inform a wide range of health care professionals including neonatologists, nurses, pharmacists and policy makers. Studies describing drug utilisation can also inform educators and guide curriculum development as they highlight the areas where medical, nursing and pharmacy curricula may need to focus.

The aim of this study was to describe patterns of drug utilisation in neonatal units in England and Wales. We aimed to identify the most frequently used drugs, the frequency at which individual drugs and groups of drugs are used, and to describe the duration of use, change in pattern and differences by gestational age (GA) and level of care.

## Methods

We performed a retrospective, descriptive, observational cohort study using de-identified, routinely recorded neonatal clinical data held in the National Neonatal Research Database (NNRD) [6]. Validation studies have shown that the completeness and quality of NNRD data are high [6]. Where infants are transferred between hospitals, episodes of care are linked to enable description of complete care. All infants admitted to neonatal units in England and Wales from 01 January 2010 to 31 December 2017 whose data are recorded in the NNRD were included. Infants with missing data on birth weight, sex or GA were excluded, and where there were discrepancies in the record, we took the entry for the first episode of care. Infants with a birth weight for GA *z* score greater than 4 or less than −4 standard deviations were excluded as improbable data. We also excluded infants with missing records from 1 or more days of care and, due to a lack of data credibility, infants born at <34 weeks’ GA who were admitted >1 day after birth, and infants born at or after 34 weeks admitted >7 days after birth.

For sub-group analyses, the cohort was categorised by gestational age at birth (extremely preterm infants, born 23–28 weeks’ gestation; very preterm, 28–31 weeks; moderate to late preterm, 32–36 weeks; term, 37–42 weeks). Units were grouped into three levels: Level 1, special care baby unit ‘SCBU’; Level 2, local neonatal unit ‘LNU’; and Level 3, neonatal intensive care unit ‘NICU’ as per their service designation [7].

The NNRD contains a daily record of the names of each drug prescribed to each infant on that day. We harmonised variations in spelling (e.g., amoxicillin and amoxycillin) and generic and brand names (e.g., Calpol® and paracetamol) in order to identify individual drugs and pharmacological groups. Several substances entered in the daily drugs record were excluded from further analyses where these were deemed to not be pharmaceuticals or where we felt their use may not be routinely recorded, including fluids such those used to maintain venous or arterial line patency (e.g., heparin sodium), standard intravenous solutions (e.g., glucose), parenteral nutrition solutions, milk formula, vitamin supplements and vaccinations.

All data management and analysis were carried out using Stata v16 (Stata Corp, College Station, TX, USA). In order to describe drug utilisation, we first calculated the median [interquartile range (IQR)] number of different drugs prescribed per infant, both overall and for four GA subgroups. We then calculated the median (IQR) number of ‘drug free days’, expressed as a percentage of the total number of days of care, and compared the characteristics of infants who received no drugs during their stay with those who received at least one drug for one day.

For each individual drug and pharmacological group, we calculated the number and percentage of infants in each GA group prescribed these for at least one day, and also the total number of days of prescribing across the whole study period. To explore variations in prescribing by neonatal unit level of care, we repeated this analysis in the subgroup of infants who were treated in just one location and who were not transferred between units.

In a post hoc analysis, we calculated the number and percentage of infants who received an antibiotic at least once, the number and percentage who received at least one course of antibiotics (defined as five consecutive days of antibiotics) and the median (IQR) number of different antibiotics prescribed.

Finally, we explored changes over time in the frequency of use of individual drugs among those born at <32 weeks’ GA. We excluded more mature infants as they are not routinely admitted to neonatal units. In addition, during the study period, data from an increasing number of near-term and term infants have been entered into the NNRD including data for those who are often admitted for short stays for observation and starting treatment for suspected sepsis and then discharge for continuing care on the postnatal wards. The inclusion of such infants in the analysis would hide true changes in patterns of prescribing among the most premature infants. For each drug, we calculated the magnitude of the difference between the calendar year with the highest prevalence of prescribing and the year with the lowest prevalence of prescribing and ranked these to identify the drugs with the largest change.

The study was registered (ClinicalTrials.gov NCT03773289) and approved by the Yorkshire & The Humber – Leeds East Research Ethics Committee (reference: 18/YH/0209).

## Results

Records of 643,233 infants admitted to 187 neonatal units across England and Wales from 01 January 2010 to 31 December 2017 were retrieved from the NNRD. After exclusions (Online Resource Fig. 1), 623,250 (97.0%) infants were included (Table 1). The total number of infants admitted increased from 2010 to 2017 and there was an increase over time in the proportion of admissions who were born at term (Online Resource Fig. 2).

**Fig. 1:**
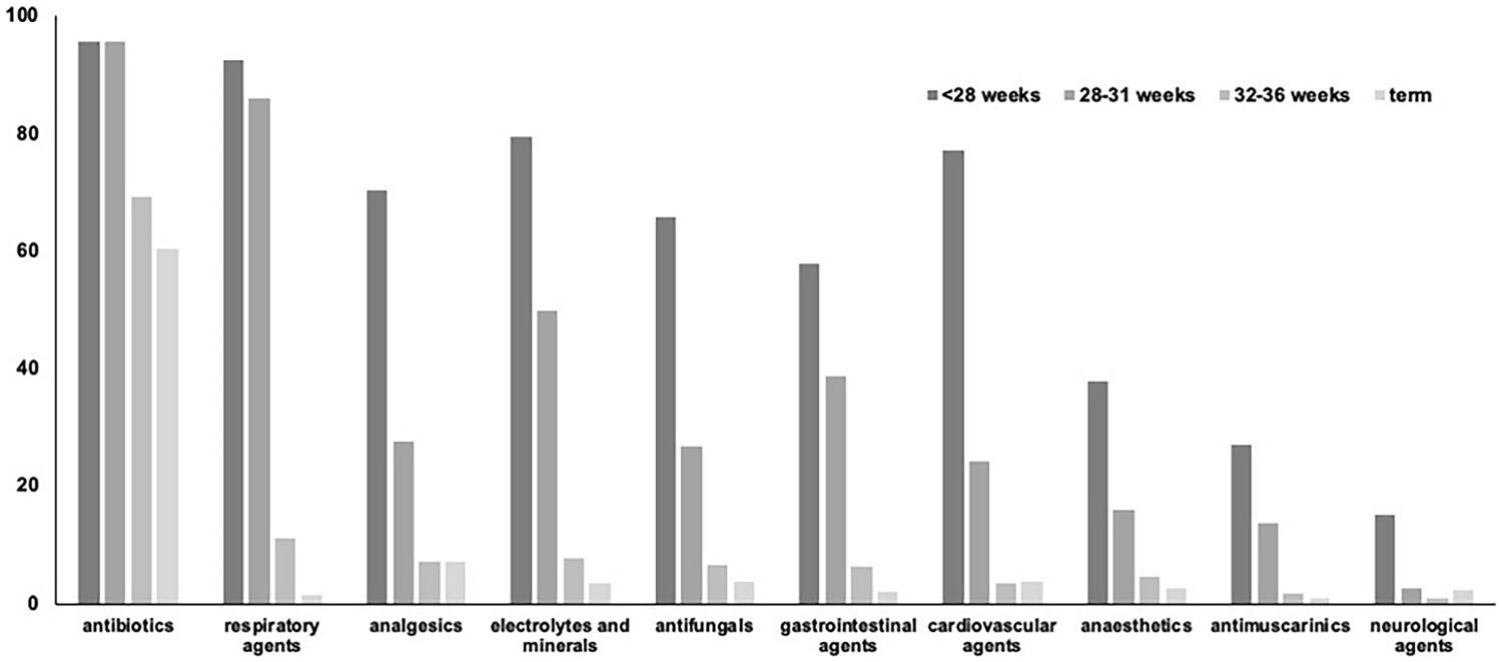
Most frequently used drug categories (British National Formulary – Children listing [7]) among newborn infants admitted to neonatal units in England and Wales (2010 to 2017)

**Table 1:** Characteristics of infants admitted to neonatal units in England and Wales from 2010 to 2017

		**All gestational age groups**	**Extremely preterm** **(< 28 weeks)**	**Very preterm** **(28–31 weeks)**	**Moderate to late preterm** **(32–36 weeks)**	**Term** **(≥ 37 weeks)**
**Number** ***n* (%)**		623,250	17,501 (2.8)	40,607 (6.5)	193,536 (31.0)	371,606 (59.6)
**Gestational age (weeks)** **median (IQR)**		37 (35–40)	26 (24–27)	30 (29–31)	35 (33–36)	39 (38–40)
**Birth weight** **(grams) median (IQR)**		2896 (2172–3500)	820 (680–970)	1380 (1170–1594)	2236 (1910–2590)	3365 (2956–3760)
**Female** ***n* (%)**		276,929 (44.4)	8,077 (46.2)	18,519 (45.6)	88,491 (45.7)	161,842 (43.6)
**Length of hospital stay (days)** **median (IQR)**		5 (3–13)	83 (58,107)	43 (33–57)	11 (5–18)	3 (2–6)
**Outcome of neonatal care, *n* (%)**	**Died**	8,378 (1.3)	4,072 (23.3)	1,309 (3.2)	1,217 (0.6)	1,780 (0.5)
	**Discharged home**	407,948 (65.5)	12,365 (70.7)	38,025 (93.6)	150,375 (77.7)	207,183 (55.8)
	**Transferred for further care**	205,326 (32.9)	1,001 (5.7)	1,213 (3.0)	41,660 (21.5)	161,452 (43.4)
	**Missing**	1,598 (0.3)	63 (0.4)	60 (0.1)	2834 (0.1)	1,191 (0.3)

*IQR*, interquartile range; *SD*, standard deviation

**Fig. 2:**
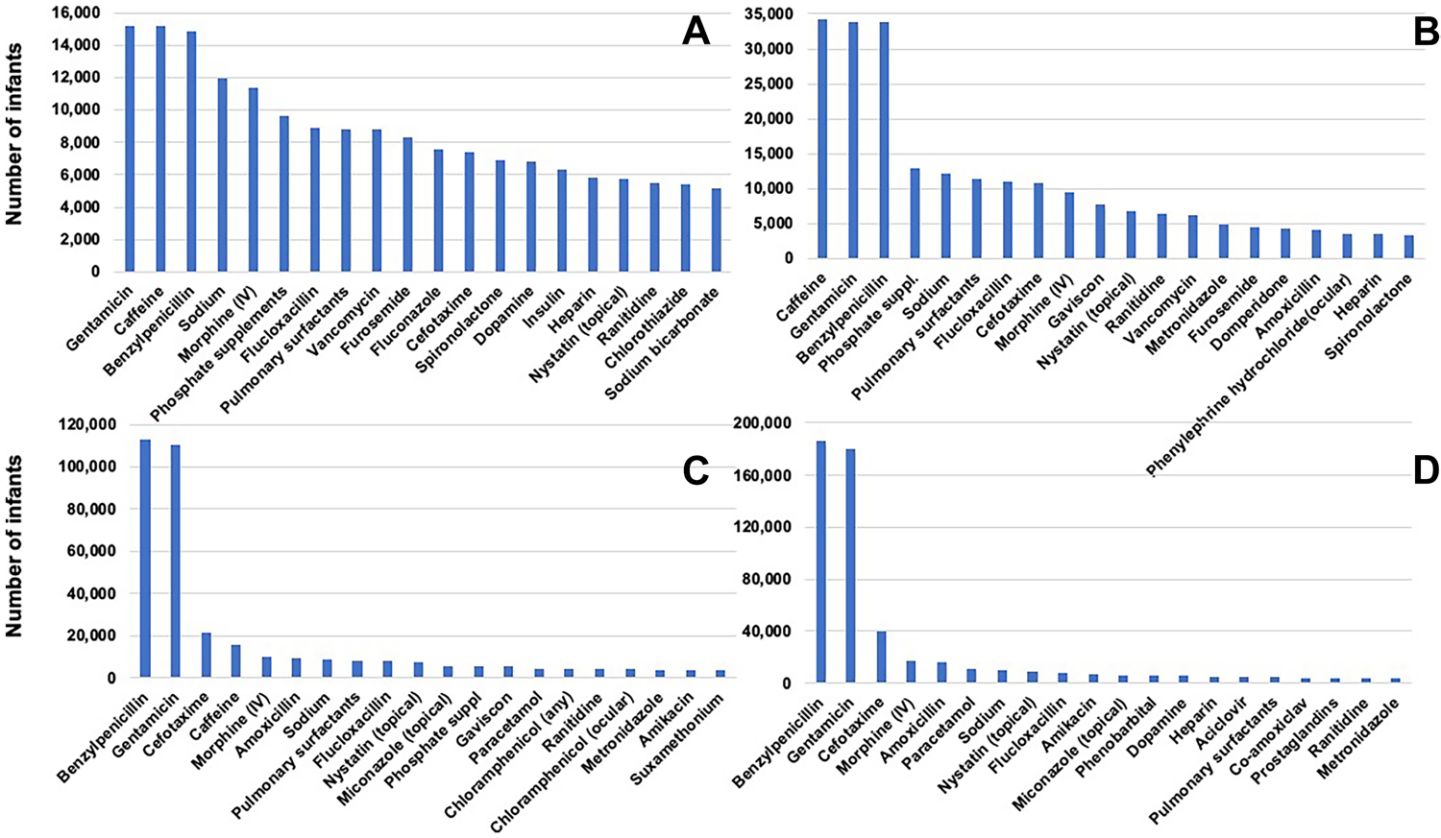
Frequency of drug use by count of number of infants who received the drug at least once among **A** extremely preterm; **B** very preterm; **C** moderate to late preterm; and **D** term infants. (Note: scale of the Y-axis varies between the panels)

After combining variations in spellings and brand names, there were 356 individual drugs which could be classified into 43 pharmacological categories as per the British National Formulary—Children listing [8]. Overall, antibiotics were the most frequently prescribed category. In the preterm subgroup, respiratory agents were the second most frequently prescribed, and analgesics were second among term infants (Fig. 1: A, extremely preterm; B, very preterm; C, moderate to late preterm; and D, term infants.).

The number of drugs prescribed per infant (median (IQR, range) was 3 (0–4, 0–73). Extremely preterm infants received the highest number of drugs, 17 (11–24, 0–73), increasing from 15 (10–22, 0–58) in 2010 to 18 (13–26, 0–57) in 2017. This was followed by the very preterm infants, 7 (5–11, 0–65); moderate to late preterm infants, 3 (0–4, 0–56); and term infants, 3 (0–3, 0–47). The term infants did not receive any of the included drugs on 40% (0–100%) of days spent in neonatal care. The percentage of ‘drug free days’ increased with increasing GA among the preterm infants (extremely preterm infants: 6% (1–18%); very preterm infants: 28% (10–50%); moderate to late preterm infants: 72% (41–100%)).

A total of 194,410 (31.2%) infants did not receive any drug during their neonatal care. Compared to those who received one or more drugs, these infants had a higher gestational age (38 (36–40) vs. 37 (34–40) weeks, *p* < 0.001) and birth weight (3000 (2040–3520) vs. 2830 (2025–3490) grams; *p* < 0.001) and spent a shorter time in neonatal care (3 (2–5) vs. 7 (3–18) days), *p* < 0.001).

## Most frequently prescribed drugs

**Fig. 3:**
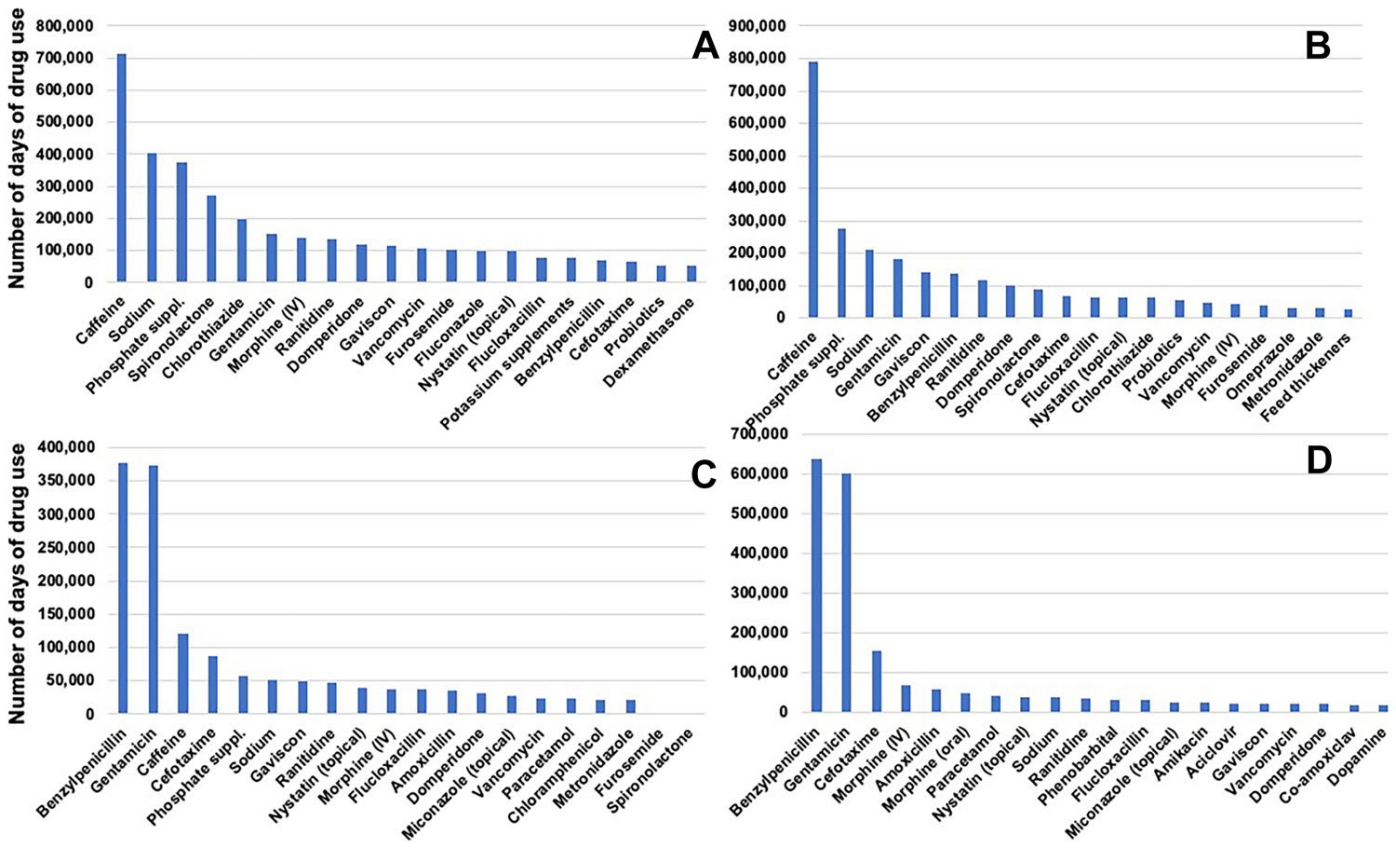
Frequency of drug use by number of days any infant received the drug among **A** extremely preterm; **B** very preterm; **C** moderate to late preterm; and **D** term infants. (Note: scale of the Y-axis varies between the panels)

Frequency of use of individual drugs was calculated in two ways: Fig. 2 panels (A, extremely preterm; B, very preterm; C, moderate to late preterm; and D, term infants) show the most frequently used drugs ranked by the number of infants who received the drug at least once and Fig. 3 panels (A, extremely preterm; B, very preterm; C, moderate to late preterm; and D, term infants) show the most frequently used drugs ranked by the total number of days of use in each GA category. Benzylpenicillin and gentamicin along with caffeine emerged as the drug given to most infants. Number of days of use of caffeine and other drugs such as sodium and phosphate supplements were higher than that of the antibiotics among the extremely and very preterm infants.

A total of 377,171 infants received all their care in one neonatal unit: 54,689 (14%) in a Level 1 unit; 168,368 (45%) in a Level 2 unit; and 154,114 (41%) in a Level 3 unit. The most frequently used drugs in each level are given in Online Resource Table 1.

## Antibiotic use

A total of 413,911 (66%) infants received at least one antibiotic during their neonatal care. Among the extremely preterm infants, 96% received at least one antibiotic and 78% received at least one course of 5 days. This most immature group was also exposed to the highest number of different antibiotics (median (IQR) 5 (3 to 6)) and received a median (IQR) 18 (9–32) days of antibiotics making up 27% (17–47) of their total neonatal care. Analyses of antibiotic usage by GA groups for infants who received at least one antibiotic are given in Table 2.

**Table 2:** Antibiotic use among infants admitted to neonatal units in England and Wales (2010–2017)

	**All gestational age groups**	**Extremely preterm** **(< 28 weeks)**	**Very preterm** **(28**–**31 weeks)**	**Moderate to late preterm** **(32**–**36 weeks)**	**Term** **(≥ 37 weeks)**
Number prescribed antibiotics at least once	423,918	17,245	40,113	136,753	228,807
Length of stay (days)	7 (1–527, 3–18)	86 (1–527, 63–110)	44 (1–419, 33–58)	13 (1–407, 6–20)	4 (1–309, 3–7)
Number of different antibiotics per infant	2 (2–2, 1–17)	5 (3–6, 1–17)	3 (2–4, 1–14)	2 (2–2, 1–12)	2 (2–2, 1–13)
Number of days on antibiotics per infants	3 (2–5, 1–305	18 (9–32, 1–305)	6 (3–12, 1–223)	3 (2–5, 1–188)	3 (2–5, 1–183)
Proportion of care days on antibiotic(s) (%)	60 (26–100, 1–100)	27 (17–47, 1–100)	15 (10–26, 1–100)	31 (18–60, 1–100)	100 (63–100, 1–100)
Number who received at least one course*	136,859	15,073	22,167	34,132	65,487
Number of courses* per infant	0 (0–1, 0–16)	2 (1–3, 0–16)	1 (0–1, 0–15)	0 (0–0, 0–10)	0 (0–1, 0–11)

All figures are median (IQR, range)

^*^Antibiotic course: antibiotics prescribed for at least 5 consecutive days. If there was a gap of ≥ 2 days between stopping and re-starting antibiotics, they were counted as two courses

## Changes in frequency of use

**Fig. 4:**
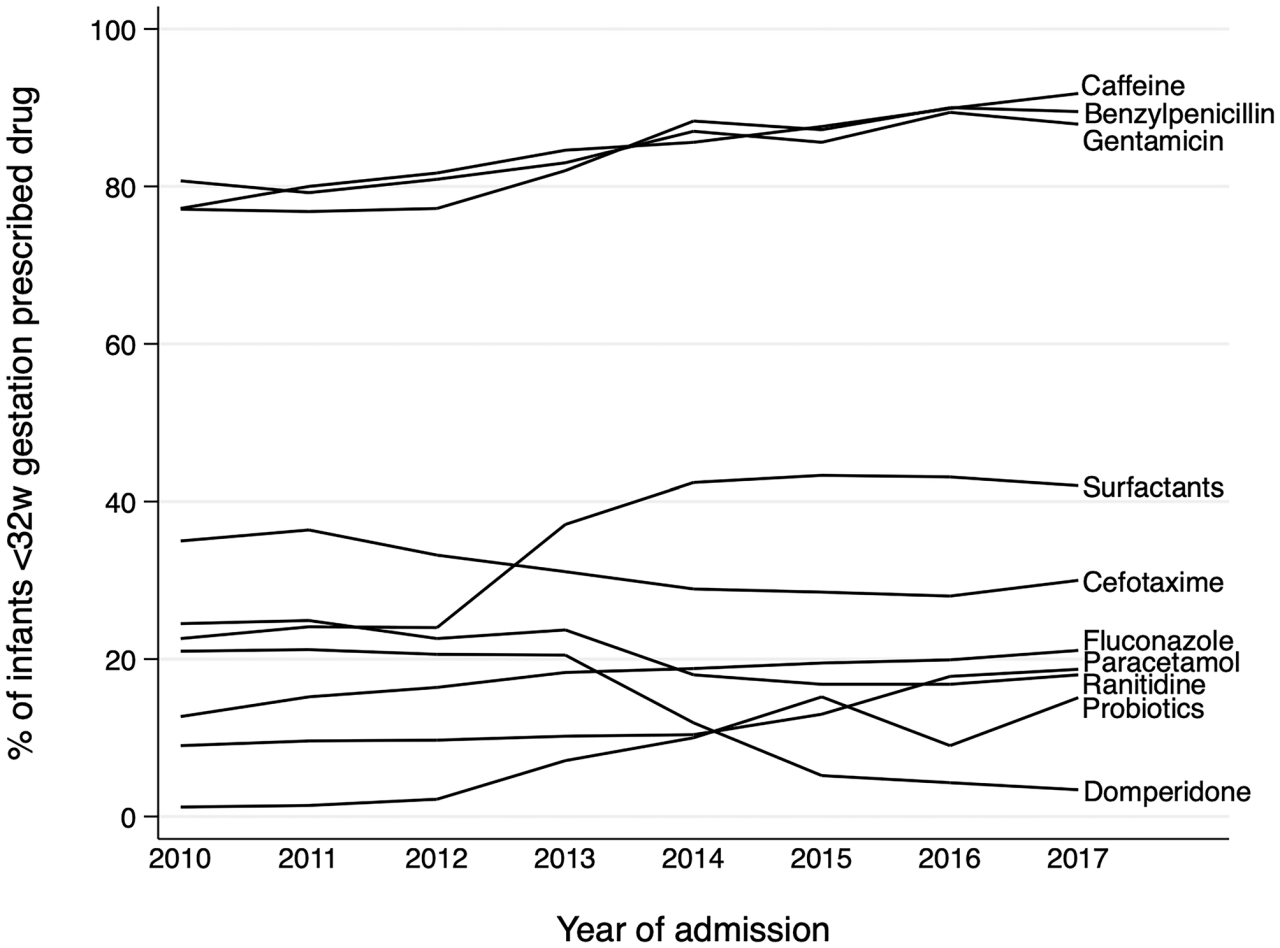
Drugs with the largest change in frequency of use among infants born at <32 weeks’ gestation in England and Wales from 2010 to 2017. (Frequency of drug use was counted as the number of infants who received the drug at least once)

The reported frequency of use of benzylpenicillin and gentamicin increased in the study period: 56% of all infants received benzylpenicillin and/or gentamicin in 2010, rising to 61% in 2017. There were increases in the frequency of prescribing in all gestational age groups (from 86% in 2010 to 93% in 2017 in extremely and very preterm infants, and from 52% in 2010 to 58% in 2017 in moderate and late preterm and term infants). The increase in the absolute numbers of term (as well as moderate to late preterm) infants over time means that, of those infants who do receive benzylpenicillin and/or gentamicin, the percentage born at the later gestations has increased over time, driving the overall increase in prescribing. Figure 4 shows changes over time in the 10 drugs with the largest change in frequency of prescribing among infants born at <32 weeks’ GA. Recorded surfactant use on the neonatal unit had the largest increase (2010: 22.6% to 2017: 42%) and domperidone had the largest decrease (2010: 21% to 2017: 3.4%). The change in drug use for all drugs with 3% or greater change is given in Online Resource Table 2.

## Discussion

This study presents a comprehensive evaluation of drug use in neonatal units in England and Wales. We found a high burden of medication exposure, especially for those born preterm. Extremely preterm infants receive, on average, 17 different drugs and spend 94% of their time on medications despite the exclusion of routine prescriptions such as multivitamins. A study from Germany reported 11 drugs per infant in two cohorts (2004 and 2014) [9]. Other studies have also reported a high burden of drug use in preterm infants. Daniell and Darlow [10] reported 14.5 drugs per infant in New Zealand while Warrier et al. reported 9.9 drugs per infant in the extremely preterm group in the USA [11] and Puia-Dumitrescu found that infants born at 22–24 weeks were exposed to 13 distinct medications [12]. Although it is difficult to compare these figures because of the wide heterogeneity of the included populations and drugs, many show an inverse relationship between GA and the number of drugs per infant [13]. Exposure to such a high number of drugs, often simultaneously and for prolonged periods, increases the risks of drug interactions and adverse reactions.

Polypharmacy and failure to adhere to evidence-based practice and clinical guidelines in starting and stopping medicines are indicators of irrational practices and it is common to administer medicines outside their authorisation [14]. Generation of high-quality evidence of drug efficacy and safety need to be combined with rational prescribing tools [15] and continuous monitoring of drug use with data that explore implementation of recommended practices [16].

No included medications were recorded for 31% of infants admitted to the neonatal units. These infants were more mature, larger and stayed in the neonatal unit for shorter periods of time compared to those who received at least one included drug. These infants are likely to be the term and near-term infants who are admitted to the neonatal unit for brief periods of observations where their clinical condition improves and therefore medications are not required.

Not surprisingly, antibiotics emerged as the most frequently used drugs and several appeared in the top ten for all GA groups. Extremely preterm infants receive, on average, 5 different antibiotics and spend nearly 30% of their care on antibiotics. Use of second- and third-line antibiotics such as cefotaxime and vancomycin has also increased. Our analyses show large increases in use of benzylpenicillin and gentamicin. A proportion of this increase is due to the larger numbers of term infants in the later years of the study who often receive antibiotics due to risk factors for early onset sepsis. The UK National Institute of Health and Care Excellence (NICE) issued guidelines for management of infants at risk of early onset sepsis in 2012 [17] which aimed to quickly treat suspected early onset sepsis and, inadvertently, led to 9% more lumbar punctures and longer durations of antibiotic treatment and hospital stay [18]. The revised NICE 2021 guidance [19] and the popular implementation of the Kaiser-Permanente sepsis risk calculator [20] have reduced antibiotic use in term and near-term infants at risk of early onset sepsis but further efforts are needed to ensure that those at risk are adequately protected while unnecessary antibiotic use is simultaneously minimised.

However, even after excluding the more mature infants, we found that the use of antibiotics has increased among those born <32 weeks’ GA. Due to their high susceptibility to infections, antibiotics are often prescribed empirically to preterm infants. Rigorous antibiotic stewardship programs tailored to the preterm population with emphasis on both reducing antibiotic initiation and shortening the duration of treatment can be effective in reducing the burden of unnecessary antibiotics in this population [21] While the benefits of antibiotic therapy, when needed, are clearly enormous, widespread use raises concerns of emergence of resistant strains, and risks of developmental and immune dysregulation with changes in gut microbiota which may have long term implications [22].

Caffeine is the preferred drug for apnoea of prematurity and was prescribed at almost the same or greater frequency as antibiotics among extremely and very preterm infants. In these groups, it was also one of the drugs given for the longest duration and its use increased during the study period. This widespread, prolonged and increase in use is likely to be the influence of evidence that demonstrated safety, efficacy and some long-term benefits [23]. However, controversies about optimal timing, dosage and duration of use remain [24] with the need for further research into optimising caffeine use.

We found that diuretics, including spironolactone and chlorthiazide, are given to most preterm infants and often for prolonged periods. Slaughter et al. reported that among <29 week infants with bronchopulmonary dysplasia in 35 USA hospitals, 86% had received diuretic therapy and although furosemide was given to most infants, chlorthiazide was given for longer duration [25]. Diuretics that act on distal tubules such as spironolactone and thiazides are less potent than loop diuretics e.g., furosemide but cause less electrolyte imbalance and hence are preferred for prolonged treatment. A few weeks’ treatment with thiazides and spironolactone can improve pulmonary mechanics; however, there is very little evidence of any sustained benefit while they can cause significant electrolyte imbalances and other adverse effects [26]. Nevertheless, our findings and other studies [25] show that diuretics remain in popular use.

Similarly, widespread use of anti-reflux medications is supported by little evidence of benefit and observational studies have shown potential for harm [27]. Santos et al. pooled results from 10 studies and found that use of H2RAs was associated with an increased odds of NEC (odds ratio (OR) 2.81, 95% confidence interval (CI) 1.19 to 6.64) and infection (OR 2.09, 95% CI 1.35 to 3.24) [28]. Gastro-oesophageal reflux disease (GORD) remains an area fraught with diagnostic and management conundrums and this high use of anti-reflux medications despite the lack of evidence for benefit and associations with harm reflects these uncertainties [29].

We found that some drugs such as domperidone decreased in use, perhaps following the evidence of lack of efficacy and associate risks of cardiac arrythmias [30]. The rapid decline is use was probably driven by alerts and the UK Medicines and Healthcare products Regulatory Agency stipulation that domperidone was no longer licensed for use in children younger than 12 years or those weighing less than 35 kg [31]. This was supported by guidance from leading national organisations such as the UK National Paediatric Pharmacists Group and the Royal College of Paediatrics and Child Health. Such whole system approaches that bring together a range of stakeholders and develop a shared understanding of the problem can bring about sustainable change in practice with direct benefit to patients. However, tacking one drug by itself is not sufficient, as in the case of anti-reflux medications, lack of continued monitoring of drug use for GORD shift in practice to avoid domperidone has led to another irrational practice to creep in i.e., the increasing use of antacids such as proton-pump inhibitors [14, 29].

Patterns of use of agents used for closure of PDA have also changed with decreased use of indomethacin and increased use of ibuprofen. Among infants born <32 weeks’ GA, we found a large increase in use of surfactant recorded in the database. While this may be due to an actual increase in use, it is possible that it represents a shift from using surfactant at delivery, which would not be recorded in this dataset, to giving surfactant after admission to the neonatal unit which would be recorded in the list of daily drugs. This change is in keeping with the increasing trend of use of non-invasive ventilation to initiate respiratory support in preterm infants [32].

There are other limitations of this study due to the manner in which data are entered into the NNRD. The database records information from all infants admitted to the unit and does not cover drugs given to infants on the postnatal wards. The drugs given each day are entered without any information on doses, regimens or method of administration. All these data would be required to assess if drug use describe here was or was not ‘rational’. The indications of use are also not defined and cannot be directly linked to diagnoses entered. Another limitation is that we excluded data on vitamin supplementation and vaccinations, both of which are vital drug groups that are frequently prescribed. We opted to exclude these drugs from the analysis because the entry of these drugs in to the NNRD is known to be inconsistent and the information available in the database was very likely to be incomplete. Despite these limitations, with the available data, this study describes the largest study of drug use in neonatal units and highlights areas from improving practice and research to optimise rational drug use.

## Conclusions

Newborn infants, especially those born preterm, receive multiple drugs, often for prolonged periods. Antibiotics are used frequently, and infants can receive several different antibiotics in the course of their care. Some changing patterns of use reflect emerging evidence, but many frequently prescribed drugs continue to be use without the evidence of benefit or lack of harm. A whole-system approach with multi-disciplinary engagement of academic, clinical, public and policy making stakeholders who work collaboratively to refine the evidence-base, provide clear guidance and embed good practices with ongoing monitoring and evaluation is required to tackle the persistence of irrational use of medicines in neonatal medicine.

## Supplementary Information

Below is the link to the electronic supplementary material.

Supplementary file1 (PDF 227 KB)

## Data Availability

The datasets generated during and/or analysed during the current study are available from the corresponding author on reasonable request.

## Code availability

Coding use for data analysis is available on request from the corresponding author.
