# Supplementary material for: Drug utilisation in neonatal units in England and Wales: a national cohort study
Source: Eur J Clin Pharmacol. 2022 Jan 13;78(4):669–77. doi: 10.1007/s00228-021-03267-x (PMC8926961; doi:10.1007/s00228-021-03267-x)

Supplementary material

Title: Drug utilisation in neonatal units in England and Wales: a national cohort study

Journal: European Journal of Clinical Pharmacology

Authors: Asma Al-Turkait[1], Lisa Szatkowski[1], Imti Choonara[1], Shalini Ojha[1,2]

[1]Academic Unit of Population and Lifespan Sciences, School of Medicine, University of Nottingham, Nottingham, UK.

[2]University Hospitals of Derby and Burton NHS Trust, Derby, UK.

Corresponding author email: shalini.ojha@nottingham.ac.uk.

Online Resource table 1. Most frequently prescribed drugs among infants who received all their care in one neonatal unit in England and Wales between 2010 and 2017.

Individual drugs	Special Care baby Units (Level 1)	Local Neonatal Units (Level 2)	Neonatal Intensive Care Units (Level 3)
Benzylpenicillin	42,215 (53.0)	131,847 (54.6)	130,023 (52.5)
Gentamicin	40,570 (51.0)	127,059 (52.6)	128,975 (52.1)
Cefotaxime	12,390 (15.6)	33,876 (14.0)	17,009 (6.9)
Caffeine	3,464 (4.4)	18,897 (7.8)	20,060 (8.1)
Sodium	2,113 (2.7)	10,401 (4.3)	14,463 (5.8)
Morphine (intravenous)	1,033 (1.3)	8,693 (3.6)	16,796 (6.8)
Amoxicillin	2,566 (3.2)	16,552 (6.9)	7,039 (2.8)
Flucloxacillin	1,915 (2.4)	9,641 (4.0)	11,336 (4.6)
Pulmonary surfactants	1,200 (1.5)	8,919 (3.7)	10,557 (4.3)
Nystatin (topical)	768 (1.0)	6,415 (2.7)	11,804 (4.8)
Phosphate supplements	629 (0.8)	6,510 (2.7)	9,564 (3.9)
Paracetamol	1,325 (1.7)	4,702 (1.9)	7,871 (3.2)
Miconazole (topical)	502 (0.6)	7,265 (3.0)	5,165 (2.1)
Gaviscon	1,787 (2.2)	6,273 (2.6)	4,208 (1.7)
Ranitidine	1,287 (1.6)	4,952 (2.1)	4,973 (2.0)
Vancomycin	268 (0.3)	2,961 (1.2)	7,624 (3.1)
Amikacin	12 (0.0)	4,109 (1.7)	6,069 (2.5)
Metronidazole	555 (0.7)	3,194 (1.3)	5,387 (2.2)
Dopamine	159 (0.2)	1,822 (0.8)	6,734 (2.7)
Chloramphenicol	1,241 (1.6)	3,527 (1.5)	3,563 (1.4)
Furosemide	256 (0.3)	2,250 (0.9)	5,489 (2.2)

Figures represent percentage of total number (%) of infants who received the drug at least once.

Online Resource table 2. Change in frequency of drug use among infants born at <32 weeks' gestation in England and Wales from 2010 to 2017.

Drug	2010	2011	2012	2013	2014	2015	2016	2017
Pulmonary surfactants	22.6	24.1	24	37.1	42.4	43.3	43.1	42
Domperidone	21	21.2	20.6	20.5	11.9	5.2	4.3	3.4
Caffeine	77.2	80	81.7	84.6	85.6	87.6	89.9	91.8
Probiotics	1.2	1.4	2.2	7.1	10	15.2	9	15.1
Benzylpenicillin	77.1	76.8	77.2	82	88.3	87.2	90	89.5
Gentamicin	80.7	79.2	80.9	83	87	85.6	89.4	87.9
Paracetamol	9	9.6	9.7	10.2	10.4	13	17.8	18.7
Cefotaxime	35	36.4	33.2	31.1	28.9	28.5	28	30
Fluconazole	12.7	15.2	16.4	18.3	18.8	19.5	19.9	21.1
Ranitidine	24.5	24.9	22.6	23.7	18	16.8	16.8	18
Nystatin (topical)	17	18.8	20.1	22.7	23	22.5	24.7	23.5
Phosphate supplements	35.8	37.2	37.3	38.9	39.9	40.2	40.1	41.8
Morphine (IV)	32.7	34	33.4	35.7	37.8	38.1	37.7	37.3
Chlorothiazide	10.5	12.2	12.7	14.1	14.6	15.7	14.7	15.3
Cyclopentolate 0.5% (ocular)	8	9	9.1	10.7	11.8	11.8	11.8	13.1
Fentanyl	2.8	4	3.9	4.1	4.3	5.5	6.1	7.8
Indomethacin	5.1	2.6	4.5	.7	.4	.6	.5	.4
Spironolactone	14.6	16.3	17.2	18.1	18.8	19.3	18.4	18.9
Phenylephrine hydrochloride 2.5% (ocular)	8.7	9.9	10.2	11.8	12.3	12.1	12.4	13.3
Suxamethonium	6.8	7.9	8	9.8	9.9	10.3	10.2	11.3
Atropine	5.3	6.7	7	8.2	7.9	8.5	8.2	9.6
Insulin	12.5	13.5	13.4	13.8	14.6	16.2	16.1	16.8
Furosemide	19.6	22	22.1	22	22.5	22.5	23.7	22.7
Nitric oxide	2.4	2.6	3.5	4.6	5.6	6.1	6.2	6.5
Amoxicillin	11.8	13.9	14.1	12.4	10.7	10.2	10.3	10.3
Omeprazole	4	4.8	4.5	4.8	4.9	5.8	6.4	7.9
Glycerol suppository	8.2	8.9	9.4	9.9	10.1	10.2	11.4	11.9
Dopamine	14.8	16.8	15.5	16.2	18.3	18.2	18.2	18
Ibuprofen	5.2	8.2	6.8	8	8.1	7.7	8.6	7.7
Metronidazole	18	19.1	18.5	18.4	16.6	15.7	16.3	16.8
Dobutamine	9.7	10.6	9.3	9.9	12	11.6	12.4	11.6
Gaviscon	22.7	22.5	21.5	20.7	20.8	20.6	19.9	19.7

Figures represent percentage of total number of infants who received the drug at least once.

Online Resource Figure 1. Infants included in the study of drug utilisation among infants admitted to neonatal units in England and Wales (2010 to 2017)

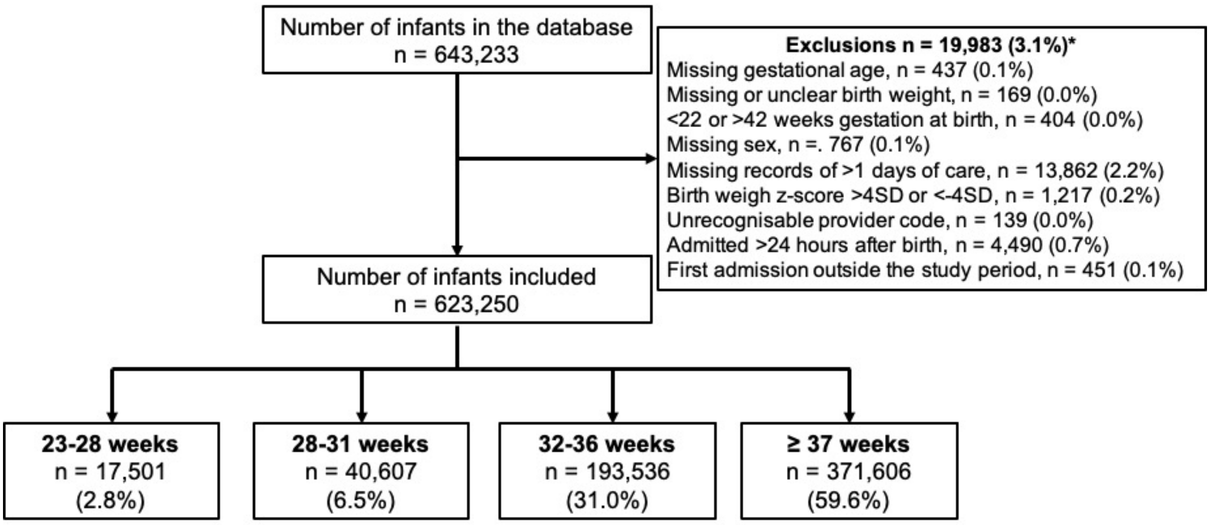

Online Resource Figure 2. Number of admissions in each gestational age at birth category among infants admitted to neonatal units in England and Wales (2010 to 2017)

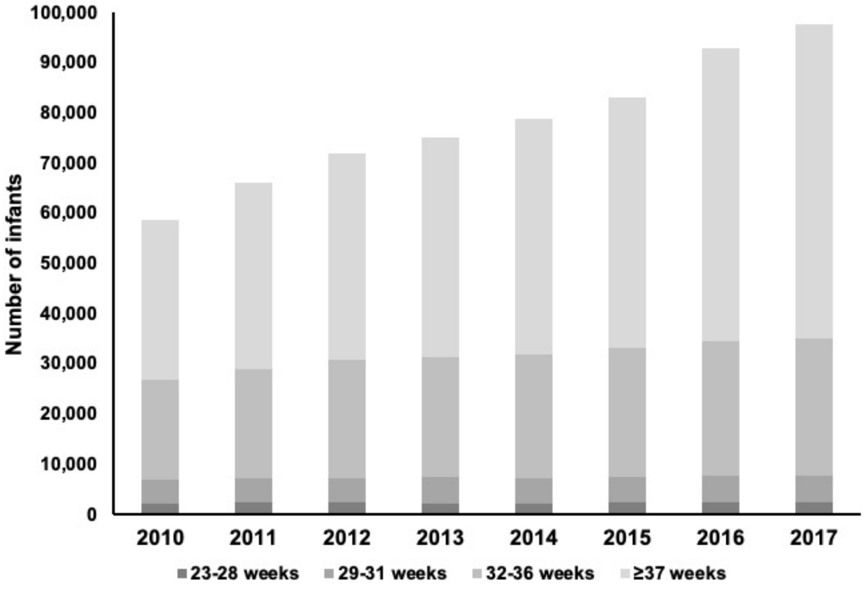

Supplement: Supplementary file 1 — Supplementary file1 (PDF 227 KB) [file 228_2021_3267_MOESM1_ESM.pdf]
